# GSK-3β inhibitor, 9-ING-41, reduces cell viability and halts proliferation of B-cell lymphoma cell lines as a single agent and in combination with novel agents

**DOI:** 10.18632/oncotarget.22414

**Published:** 2017-11-11

**Authors:** Reem Karmali, Vineela Chukkapalli, Leo I. Gordon, Jeffrey A. Borgia, Andrey Ugolkov, Andrew P. Mazar, Francis J. Giles

**Affiliations:** ^1^ Division of Hematology/Oncology, Northwestern University Feinberg School of Medicine, Chicago, IL, USA; ^2^ Robert H. Lurie Comprehensive Cancer Center of Northwestern University, Chicago, IL, USA; ^3^ Department of Hematology, Oncology and Stem Cell Therapy, Rush University Medical Center, Chicago, IL, USA; ^4^ Departments of Pathology and Cell & Molecular Medicine, Rush University Medical Center, Chicago, IL, USA; ^5^ Center for Developmental Therapeutics Northwestern University, Evanston, IL, USA; ^6^ Division of Hematology/Oncology, Northwestern University Feinberg School of Medicine, Chicago, IL, USA; ^7^ Department of Pharmacology, Northwestern University Feinberg School of Medicine, Chicago, IL, USA; ^8^ Developmental Therapeutics Consortium, Chicago, IL, USA

**Keywords:** GSK-3β inhibitor, DLBCL, Myc+ Lymphoma, Bcl-2 inhibitor, CDK9 inhibitor

## Abstract

The complexities of GSK-3β function and interactions with PI3K/AKT/mTOR signaling, cell cycling, and apoptotic pathways are poorly understood in the context of lymphomagenesis and cancer therapeutics. In this study, we explored the anti-tumor effects of the GSK-3β inhibitor, 9-ING-41, in lymphoma cell lines as a single agent and in combination with novel agents comprising BCL-2 inhibitor (Venetoclax), CDK-9 inhibitor (BAY-1143572) and p110δ-PI3K inhibitor (Idelalisib). Treatment of Daudi, SUDHL-4, Karpas 422, KPUM-UH1, and TMD8 lymphoma cell lines with 1 μM 9-ING-41 reduced cell viability by 40-70% (p<0.05) and halted proliferation. Luminex analysis of apoptotic pathways revealed a significant increase in active caspase 3 in all lymphoma cell lines (p<0.001) except TMD8 cells. Co-treating SUDHL-4 and KPUM-UH1 lymphoma cells with 0.5 μM 9-ING-41 showed 8-and 2-fold reduction in IC_50_ values of Venetoclax, respectively. No significant benefit for this combination was seen in other lymphoma cells tested. The combination of BAY-1143572 with 0.5 μM 9-ING-41 showed an 8-fold reduction in the IC_50_ value of the former in SUDHL-4 lymphoma cells alone. No significant changes in IC_50_ values of Idelalisib were measured across all cell lines for the combination of 9-ING-41 and Idelalisib. Further, signaling analysis via Western blot in the double-hit lymphoma cell line, KPUM-UH1, suggests that phospho-c-MYC is modified with 9-ING-41 treatment. Altogether, our data show that 9-ING-41 results in increased apoptosis and decreased proliferation in aggressive B-cell lymphoma cells and enhances the antitumor effects of BCL-2 and CDK-9 antagonists.

## INTRODUCTION

The clinical course for diffuse large B-cell lymphoma (DLBCL) remains variable despite improved response and survival with the addition of the anti-CD20 monoclonal antibody, rituximab, to standard chemotherapy in the late 1990’s [1, 2]. Although 60% of patients enjoy long-term disease-free survival, a subset of patients with adverse biology will have chemotherapy-refractory disease with less favorable outcomes [1, 3]. In particular, dual translocation of c-*MYC* and *BCL-2* in DLBCL, termed “double hit lymphoma” (DHL), is associated with poor outcomes following standard R-CHOP (rituximab, cyclophosphamide, doxorubicin, vincristine, and prednisone), with few patients achieving long-term survival [4].

Glycogen synthase kinase-3 (GSK-3) is a serine (S) /threonine (T) kinase initially described as a key regulator of metabolism, specifically glycogen biosynthesis [5]. It has since been shown to play a role in several disease processes, including cancer and aging, immune disorders, metabolic disorders, and neurological disorders through modulation of a large and diverse number of substrates [6–10]. GSK-3 has two ubiquitously expressed and highly conserved isoforms, GSK-3α and GSK-3β, with both shared and distinct substrates and functional effects.

**Figure 1:**
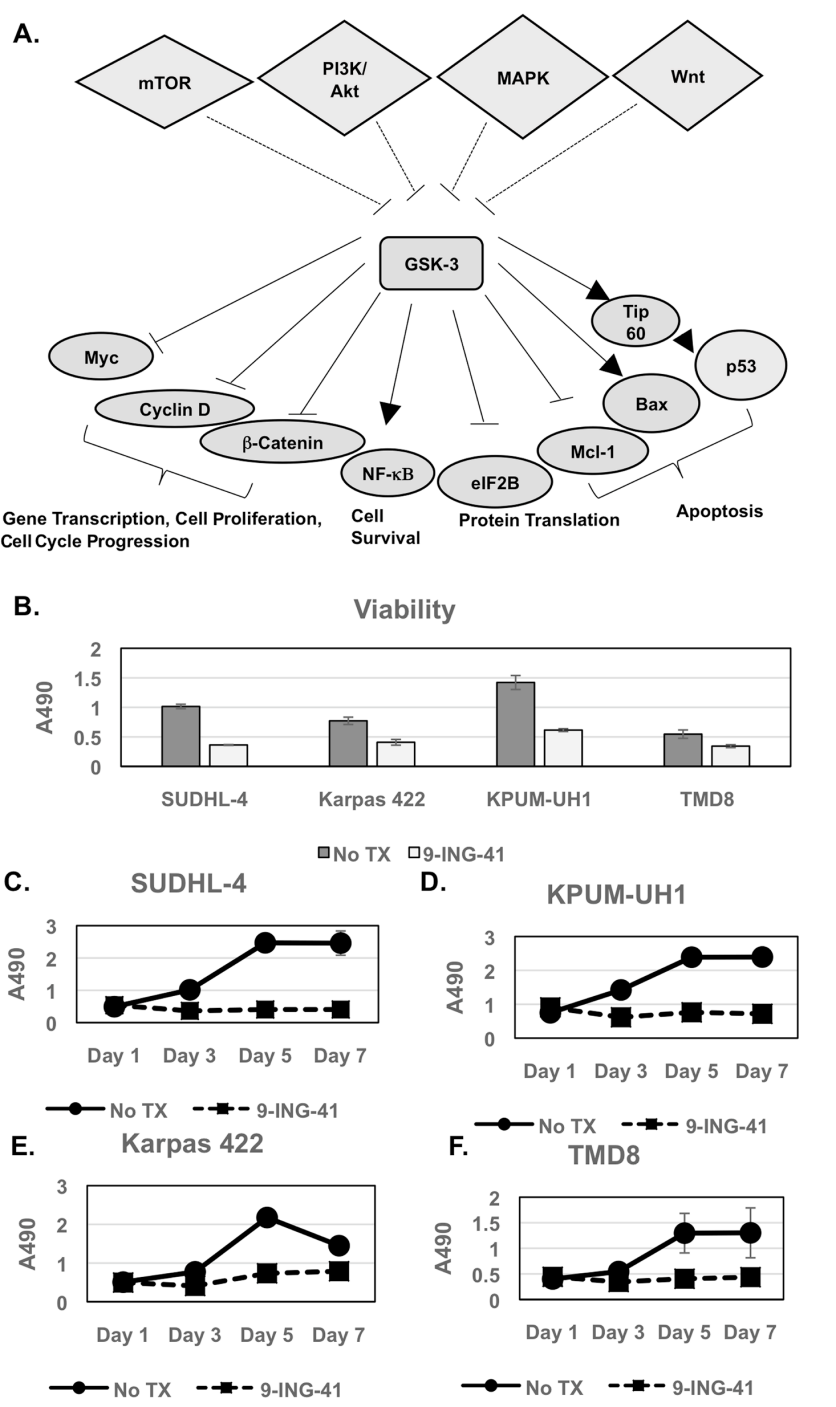
Viability and proliferation of lymphoma cells with 9-ING-41 treatment **(A)** Schematic representing GSK-3 up-stream regulators and down-stream targets and their role in cellular processes in cells. It should be noted that several of the target proteins are misregulated in cancer cells. Only target proteins relevant for this paper are shown for simplicity. **(B-F)** 10,000 cells (SUDHL-4, KPUM-UH1, Karpas 422, TMD8) per 96-well plate well were left untreated or treated with 1μM 9-ING-41 in triplicate, and the number of cells on days 1, 3, 5 and 7 were calculated using the MTS assay. Briefly, 20μL of MTS reagent was added to cells and incubated for 2 hours and, the absorbance at 490 nm (A490) was read using a Biotek plate reader. Error bars represent std. deviation between replicates. Day 3 viability is shown in B.

In cancer, much focus has been placed on the role of GSK-3β in tumor progression and modulation of oncogenes (beta-catenin, cyclin D1, and *c-MYC*), cell cycle regulators (e.g., p27^Kip1^), and mediators of epithelial-mesenchymal transition (e.g., snail) by GSK-3β have been described (Figure 1A) [11–15]. More recently, aberrant overexpression of GSK-3β has been shown to promote tumor growth and chemotherapy resistance in various solid tumors, including pancreatic, ovarian, colon cancer, and glioblastoma [16–20], through differential effects on pro-survival nuclear factor (NF)-κB and c-MYC pathways as well on tumor necrosis factor-related apoptosis-inducing ligand (TRAIL) and p53-mediated apoptotic mechanisms (Figure 1A) [21, 22]. GSK-3β is thus emerging as a potential therapeutic target in solid tumors.

By contrast, little is known about the significance of GSK-3β in B-cell lymphoma pathogenesis, resistance to therapy, and survival despite its known function as a metabolic checkpoint regulator in B-cells [23]. The effects of GSK-3β across various histologic subtypes of aggressive B-cell lymphomas and according to *c-MYC* status have yet to be explored. Herein, we explore mechanisms of anti-lymphoma activity of the GSK-3β inhibitor 9-ING-41 and address the feasibility of targeting GSK-3β in lymphoma as a single agent. We also explore the effects of this inhibitor in combination with novel agents, including the BCL-2 inhibitor (Venetoclax), CDK-9 inhibitor (BAY-1143572), and p110δ-PI3K inhibitor (Idelalisib), as a means of uncovering complimentary anti-tumor pathways that can be targeted.

## RESULTS

### 9-ING-41 treatment of lymphoma cells reduces viability and halts proliferation

All lymphoma cell lines used in this study express active GSK-3β, as shown in Supplementary Figure 1. SUDHL-4, KPUM-UH1, Karpas 422, or TMD8 lymphoma cells were plated, and cell numbers on days 1, 3, 5 and 7 were measured using the MTS assay as described in Methods. Cell viability on day 3 (Figure 1B) was reduced 40-70% (p<0.05) upon 1 μM 9-ING-41 treatment, with SUDHL-4 and KPUM-UH1 showing the highest reduction in cell viability. Upon exposure to 1μM 9-ING-41, all lymphoma cell lines underwent growth arrest (Figure 1C-1F) with proliferation of less than 30% on day 7, relative to control (p<0.05). Cell viability of lymphoma cells with varying concentrations of 9-ING-41 (0.1 μM, 0.5 μM, 1 μM, 5 μM, and 10 μM) was also tested (Supplementary Figure 2A), and a reduction in viability was only seen at concentrations of 9-ING-41 that were 0.5 uM or higher. Similarly, using the ENZCHEK Caspase 3 assay kit, there was an increase in observed Caspase 3/7 activity when lymphoma cells were treated with 0.5 μM or higher concentrations of 9-ING-41 (Supplementary Figure 2B). Previous pharmaco-kinetic studies in Xenograft mice suggest that 20 mg/kg intra-venous administration can provide around 8 μM 9-ING-41 concentration in plasma and around 40 μM 9-ING-41 in the brain within 30 minutes, suggesting that 1 μM is probably an achievable dose *in vivo* [24]. Altogether, these data suggest that 9-ING-41 inhibits proliferation of lymphoma cell lines as a single agent and reduces viability of lymphoma cells via induction of apoptosis.

### Signaling changes associated with 9-ING-41 treatment are cell-line dependent

**Figure 2:**
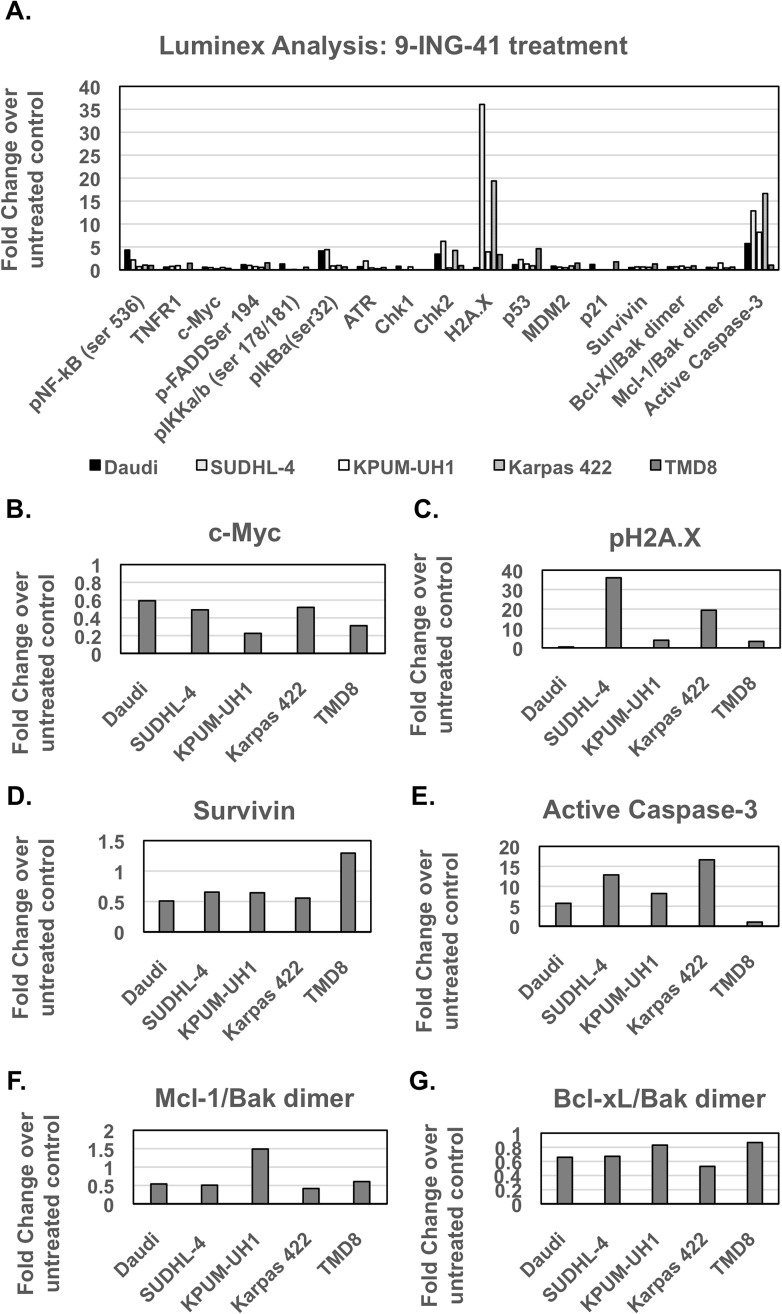
Luminex signaling analysis of lymphoma cells treated with 9-ING-41 (Related to Table 1) **(A)** 1 million cells were left untreated or treated with 1μM 9-ING-41 for 48 hrs and were then lysed in Millipore MAP lysis buffer. After protein concentration determination via BCA, an equal amount of protein around 10-15 μg (depending on the kit) of protein was used for luminex analysis. Samples were run in duplicates and according to manufacturer instructions. After setting the net MFI or absolute quantity of untreated control to 1 for each analyte, the fold change with 9-ING-41 treatment was calculated (>1 is an increase in levels, <1 is a decrease in levels). Analytes that show significant changes are shown as individual panels. **(B)** c-Myc, **(C)** pH2A.X, **(D)** Survivin, **(E)** Active Caspase 3, **(F)** Mcl-1/Bak Dimer, **(G)** Bcl-Xl/Bak dimer.

Daudi, SUDHL-4, KPUM-UH1, Karpas 422, and TMD8 cells (10^7^ cells per condition) were left untreated or treated with 1 μM 9-ING-41 for 48 hours and then were lysed and analyzed as described in the Methods section. Analysis of NF-κB signaling showed a significant reduction in total c-MYC levels in Karpas 422 and TMD8 cell lines but only trends for reduction in this protein in the remaining cell lines (Table 1, Figure 2A/2B). In addition, analysis of S62-phosphorylated c-MYC showed multiple isoforms in 9-ING-41-treated cells, which is suggestive of extensive transcriptional or post-translational processing of c-MYC and which needs further investigation (Supplementary Figure 3). DNA damage signaling, via evaluation of p-H2A.X (Ser139), was found to be increased for SUDHL-4 and Karpas 422 cell lines (both p<0.05) (Table 1, Figure 2A/2C). In addition, a significant increase in phospho-p53 (Ser15) upon 9-ING-41 treatment was observed only in SUDHL-4 and TMD8 cells. Apoptosis signaling pathway analysis revealed a significant reduction (∼2-fold, p<0.05) in survivin and an increase in active caspase 3 in all lymphoma cell lines except TMD8 (Table 1, Figure 2A/2D/2E). With the exception of KPUM-UH1, all lymphoma cell lines showed significant reductions (∼2-fold, p<0.05) in Mcl-1/Bak dimers, while Bcl-xl/Bak dimer expression was significantly (∼1.5-fold, p<0.05) reduced in all cell lines except TMD8 (Table 1, Figure 2A/2F/2G). Of note, although the TMD8 cell line did not show active caspase 3 in luminex analysis, analysis for caspase 3 and closely related proteases activity via the EnzChek caspase 3/7 assay kit revealed an increase upon 9-ING-41 treatment, suggesting that other proteases/caspases might be relevant for this cell line (data not shown). Thus, 9-ING-41 treatment-induced signaling is cell-type dependent and mainly impacts c-MYC levels and apoptosis signaling in all cell-lines and induces DNA damage signaling in all cell lines tested with the exception of Daudi cells.

**Table 1:** Luminex Analysis of NF-κB signaling, DNA damage, and apoptotic pathways associated with exposure to 1 μM 9-ING-41

	p-NFκB (Ser 536)	TNFR1	c-MYC	p-FADD (Ser194)	p-IKKα/β (Ser177/181)	pIκBα (Ser32)	ATR	pChk1 (Ser 345)	pChk2 (Thr 68)	pH2A.X (Ser139)	pP53 (Ser15)	MDM2	p21	Survivin	Bcl-xL/Bak dimer	Mcl-1/Bak dimer	Active caspase 3
**SUDHL-4**	2.1 (0.05)	0.7 (0.67)	0.4 (0.38)	0.9 (0.93)	0.06 (0.19)	4.4 (0.01)	1.9 (0.63)	ND (0.25)	6.2 (0.04)	**36 (0.006)**	2.2 (0.01)	0.6 (0.62)	ND (0.45)	**0.6 (0.01)**	**0.6 (0.001)**	**0.5 (0.008)**	**12.8 (8.24E-05)**
**Daudi**	4.3 (0.01)	0.6 (0.48)	0.5 (0.29)	1.1 (0.82)	1.29 (0.51)	4.1 (0.06)	0.6 (0.01)	0.7 (0.65)	3.4 (0.23)	0.4 (0.48)	1.1 (0.18)	0.8 (0.03)	1.1 (0.33)	**0.5 (0.003)**	**0.6 (0.005)**	**0.5 (0.01)**	**5.7 (0.0003)**
**KPUM-UH1**	0.7 (0.66)	0.9 (0.89)	0.2 (0.13)	0.7 (0.66)	0.08 (0.28)	0.8 (0.83)	0.4 (0.24)	0.6 (0.50)	0.4 (0.63)	3.9 (0.14)	1.2 (0.92)	0.4 (0.12)	ND (0.06)	**0.6 (0.01)**	**0.8 (0.02)**	1.4 (0.18)	**8.1 (0.001)**
**Karpas 422**	1.0 (0.92)	ND (0.05)	**0.5 (0.02**)	0.5 (0.08)	ND (0.08)	0.9 (0.90)	0.2 (0.07)	ND (0.06)	4.2 (0.002)	**19.3 (0.02)**	0.8 (0.76)	0.8 (0.51)	ND (0.11)	**0.5 (0.04)**	**0.5 (0.01)**	**0.4 (0.01)**	**16.6 (0.0009)**
**TMD8**	0.9 (0.78)	1.4 (0.52)	**0.3 (0.03)**	1.5 (0.09)	0.56 (0.10)	0.6 (0.03)	0.5 (0.02)	ND (0.25)	0.9 (0.84)	3.3 (0.15)	4.6 (0.002)	1.4 (0.16)	1.7 (0.008)	1.2 (0.09)	0.8 (0.18)	**0.6 (0.02)**	1 (0.41)

Relative amount shown in table (Vehicle control set to 1, >1= increase,<1=decrease). p-value in commas.

ND not determined due to –ve MFI either for the ctrl or for the treatment condition but the p-values are still shown.

### 9-ING-41 enhances the antitumor effects of BCL-2 inhibitor Venetoclax and CDK9 inhibitor BAY-1143572 in a cell-type dependent manner

**Figure 3:**
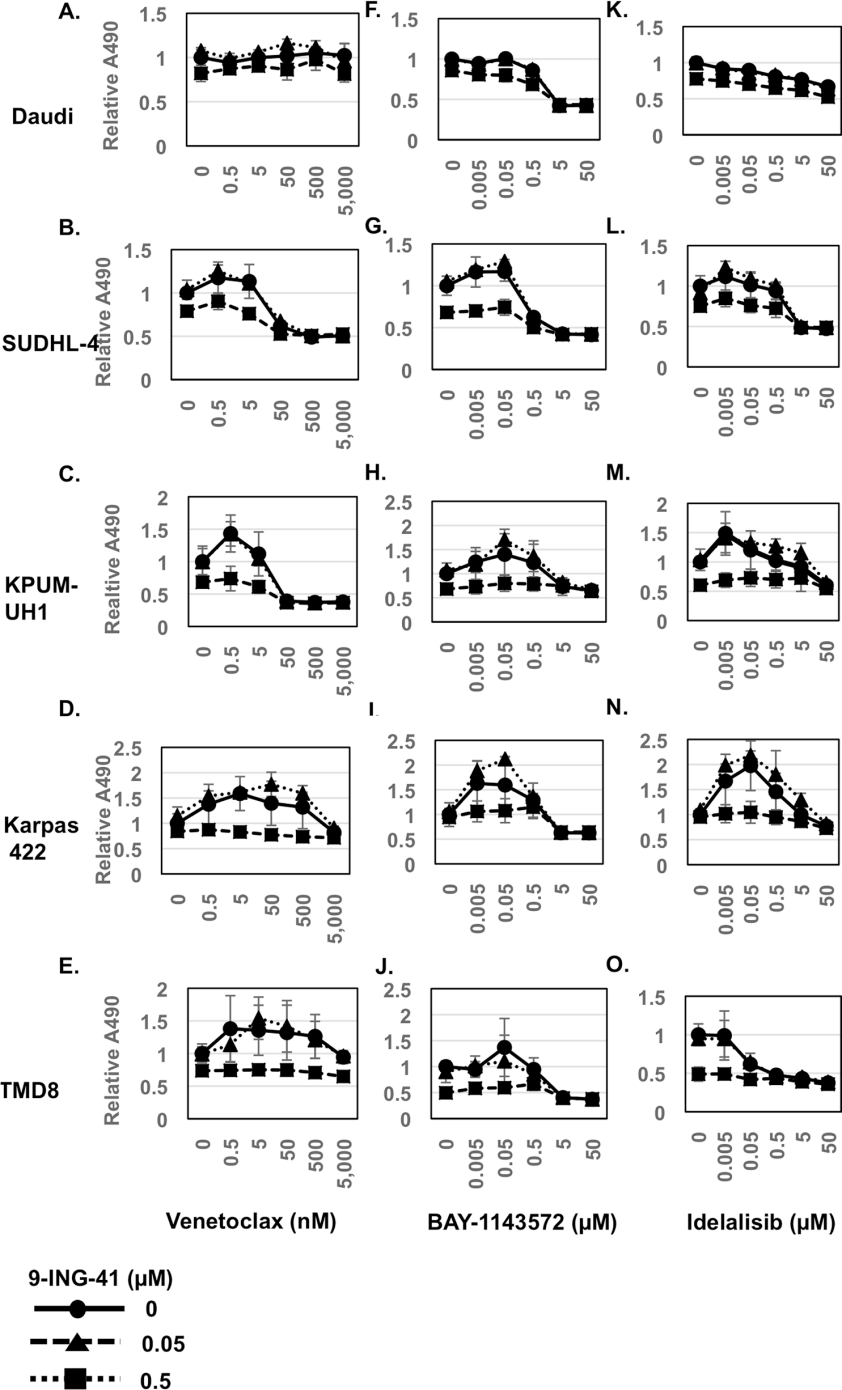
Viability of lymphoma cells with novel chemotherapy agents in combination with 9-ING-41 (Related to Table 2) 10,000 cells (A, F, K: Daudi; B, G, L: SUDHL-4; C, H, M: KPUM-UH1; D, I, N: Karpas 422; E, J, O: TMD8) were plated per well of a 96-well plate and treated with a dose response series of both 9-ING-41 (0-0.5 μM) and Venetoclax (0-5,000 nM) **(A-E)** or BAY-1143572 (0-50 μM) **(F-J)** or Idelalisib (0-50 μM) **(K-O)** in triplicate. Viability after 3 days was analyzed using the MTS assay. Briefly, 20μl of MTS reagent was added to cells and incubated for 2 hours and, the absorbance at 490 nm was read using a Biotek plate reader. Relative absorbance is calculated after setting the average absorbance of the no-treatment control as 1.

To determine if 9-ING-41 augments activity of novel targeted drugs, we tested the combination of 9-ING-41 with either the BCL-2 inhibitor Venetoclax, the CDK9 inhibitor BAY-1143572, or the PI3K inhibitor Idelalisib. Day 3 viability was determined using the MTS assay (Figure 3). Changes in IC_50_ values with combinatorial strategies are summarized in Table 2. Co-treating SUDHL-4 and KPUM-UH1 cells with 0.5 μM 9-ING-41 showed 8-fold and 2-fold reduction in IC_50_ values of Venetoclax, respectively. The remaining cell lines were insensitive to Venetoclax. The combination of BAY-1143572 with 0.5 μM 9-ING-41 also showed an 8-fold reduction in the IC_50_ value of the former in SUDHL-4 cells, but no significant benefit was observed in other cell lines. For the combination of 9-ING-41 and Idelalisib, no significant changes in the IC_50_ values of Idelalisib were measured across all cell lines.

**Table 2:** IC_50_ concentrations for novel targeted agents +/− 0.5 μM 9-ING-41

	Daudi	SUDHL-4	KPUM-UH1	Karpas 422	TMD8
**Venetoclax**	NR	**∼400 nM**	**∼40 nM**	NR	NR
**Venetoclax + 9-ING-41**	NR	**∼50 nM**	**∼25 nM**	NR	ND
**BAY1143572**	∼5 μM	**∼4 μM**	NR	NR	∼4.5 μM
**BAY1143572 + 9-ING-41**	∼4 μM	**∼ 0.5 μM**	NR	NR	ND
**Idelalisib**	NR	∼5 μM	NR	NR	∼0.5 μM
**Idelalisib + 9-ING-41**	NR	∼5 μM	NR	NR	ND

NR= not-reached; ND=not-determined (9-ING-41 as a single agent was more effective).

In order to better understand the signaling changes occurring in 9-ING-41-treated lymphoma cells, we focused on the double-hit lymphoma cell line (KPUM-UH1), which is typically chemo-resistant. c-MYC levels were analyzed in KPUM-UH1 cells via western blot for 9-ING-41 alone and the combination of 9-ING-41 with either Venetoclax or BAY-1143572 (Supplementary Figures 3, 4). 9-ING-41 treatment alone reduced total c-MYC protein expression but showed high-molecular weight modified p-c-MYC (S62) and p-c-MYC (Thr 58) (Supplementary Figures 3, 4). Venetoclax, but not BAY-1143572, reduced c-MYC levels substantially for this cell-line, and any added benefit with 9-ING-41 with this combination was hard to visualize. The identity of the c-MYC modifications in 9-ING-41-treated cells and signaling changes that are altered with the combinations of drugs in all cell-lines are being explored further.

## DISCUSSION

We investigated the effects of the single agent 9-ING-41 on viability and proliferation in B-cell lymphoma cell lines of varying histology and biology. We also explored the effect of 9-ING-41 used in combination with chemotherapy and the novel targeted agents, Venetoclax, BAY-1143572, and Idelalisib

Our results show that GSK-3β targeting leads to decreased proliferation and viability of all aggressive B-cell lymphoma cell lines tested by inducing variable effects on pro-survival signals and DNA damage response, ultimately leading to apoptosis. In DLBCL cell lines, these effects were independent of cell of origin. The activity of 9-ING-41 in the DHL cell line KPUM-UH1, a typically chemotherapy-resistant cell line, is of particular interest. Mechanistically, our Luminex data suggest that 9-ING-41 exerts effects in this particular cell line through down-regulation of c-MYC signaling and induction of apoptosis through reduction of survivin. Down-regulation of survivin, however, does not appear to be associated with, or driven by, changes in NF-κB in this cell line. This is unlike what has been described in acute lymphoblastic leukemia (ALL) where GSK-3β suppression sensitizes ALL cells to NF-κB-mediated apoptosis via survivin effect [25].

Additive effects were not seen with the combination of 9-ING-41 and the PI3K inhibitor Idelalisib. Cross-regulation of the PI3K/PTEN/Akt/mTOR and GSK-3β has been described and is possibly occurring based on the data presented [26]. In fact, inhibition of GSK-3β represents a pathway of resistance in lymphomas preferentially dependent on PI3K for proliferation consistent with findings by Dal Col, et al. [26].

By contrast, when 9-ING-41 was combined with either Venetoclax or BAY-1143572, it increased sensitivity of KPUM-UH1 and SUDHL-4 cells to the BCL-2 inhibitor and SUDHL-4 cells to the CDK9 inhibitor, respectively. As such, effects of these combinations appear to be more pronounced in DLBCL cell lines of germinal center origin.

Venetoclax was recently approved for relapsed/refractory chronic lymphocytic leukemia patients with 17p deletion [27]. However, its activity as a single agent in non-Hodgkin Lymphoma has been quite variable. In a Phase I trial of a 106 patients (MCL; n=28, FL; n=29, DLBCL; n=34), overall response rate was only 44% (MCL, 75%; FL, 38%; DLBCL, 18%), with an estimated median progression-free survival of 6 months, requiring doses as high as 800 mg in MCL and 1200 mg in the DLBCL for efficacy [28]. Strategies that could potentiate the anti-tumor effects of Venetoclax are of interest, with 9-ING-41 serving as an attractive combination partner. In fact, in DHL, a cooperative group exploring DA-EPOCH-R + Venetoclax is underway (NCT03036904). We would reason that 9-ING-41 combined with Venetoclax is a rational approach for this patient population in the relapsed/refractory setting.

The specificity of additive effects of 9-ING-41 and the CDK9 inhibitor BAY-1143572 to one cell line, SUDHL-4, is reflective of the complexities of GSK-3β interactions with cell cycling pathways in hematologic malignancies. For example, in myeloma, selective GSK-3 inhibition has been shown to antagonize the effects of a CDK9-7 inhibitor, resulting in the rescue of myeloma cell survival [29]. Further exploration of the biologic mechanisms of response versus no response to this combination is warranted in lymphoma. As a limitation of our data, western blots and/or proteomics were not performed in detail for combination experiments with agents, with the mechanisms of additive effects remaining unclear.

To summarize, our study is the first to explore the relevance of GSK-3β inhibition as a therapeutic strategy in aggressive B-NHL with promising activity noted in DHL. We show that 9-ING-41 has significant single agent activity with the ability to induce apoptosis through variable mechanisms in a cell line-dependent manner. In our DHL cell line, 9-ING-41 is effective as a single agent and has an additive effect with the BCL-2 inhibitor, Venetoclax. 9-ING-41 also demonstrated additive effects with the CDK9 inhibitor BAY-1143572 in a germinal center cell line. We recognize that the beneficial effect of 9-ING-41 with. Venetoclax or BAY-1143572 in relevant lymphoma cell lines may be minimal but contend that our data support the need for ongoing investigation of GSK-3β as a therapeutic target in single and combinatorial strategies. We expect that combination therapy would enable lower dosing of each targeted drug with the benefit of avoiding adverse effects associated with higher doses of each agent. Alternatively, 9-ING-41 may sensitize resistant cells to these novel agents, expanding their use across a broader clinical spectrum.

## MATERIALS AND METHODS

### Cell lines

Daudi (Burkitt) and SUDHL-4 (germinal center (GC) diffuse large B-cell lymphoma (DLBCL)) cell lines were purchased from American type; culture; collection. (ATCC), whereas KPUM-UH1 (double hit DLBCL) cells were a kind gift from Junya Kuroda, Kyoto Prefectural University of Medicine, Kyoto, Japan. All cell lines were cultured aseptically and maintained in a water-jacketed incubator (Thermo-Forma) at 37°C with 5% CO_2_ and fed with RPMI-1640 (Corning) containing 0.3 g/mL glutamine, 10% FBS (Sigma), and antibiotic and antimycotic reagent (Gemini Bioproducts; final concentrations - 100 units/mL penicillin G, 100 μg/mL streptomycin sulfate, and 250 ng/mL amphotericin B). Karpas 422 (GC-DLBCL) cells was purchased from Sigma, and TMD8 (Activated B-Cell (ABC) DLBCL) cells were a gift from the lab of Dr. Louis Stoudt from the NCI and were maintained as above, but with 20% FBS. All cell numbers for assays were quantified using a TC20 automated cell counter (BioRad). All lymphoma cell lines tested express active GSK-3β (Supplementary Figure 1).

### Drugs

9-ING-41 was a kind gift from Actuate Therapeutics. Venetoclax and Idelalisib were purchased from Selleck Chemicals. BAY-1143572 was purchased from Active Biochem. All drugs were resuspended in DMSO. The no-treatment control mentioned in the manuscript is a DMSO-control with no drugs.

### Viability and proliferation assays

Cell viability at day 3 and proliferation over the course of 7 days were measured after treating cells with varying concentrations of 9-ING-41 using the Promega CellTiter 96^®^ AQ_ueous_ One Solution Cell Proliferation Assay reagent (MTS), as per manufacturer instructions. Briefly, at the end of the treatment, 20 μL of reagent was added per well in a 96-well plate and incubated for 2-4 h at 37°C. The absorbance at 490 nm (A490) was determined using a Powerwave XS plate reader (Biotek).

### EnzChek® caspase 3 assay

The EnzChek caspase 3 assay (Thermo Fisher Scientific) was performed as per manufacturer’s instructions. Briefly, 100,000 cells were plated in a 12-well plate and treated with varying concentrations of 9-ING-41 for 24 hours in duplicate. At the end of treatment, cells were centrifuged at 200 rcf for 5 minutes and washed once with 1X PBS and lysed in 50 μL of 1X lysis buffer provided by the kit. For efficient lysis, cells were subjected to a single freeze-thaw cycle. The lysed cells were centrifuged again to remove cell debris, and the supernatant was used in the assay. 50 μL of 2X substrate working solution containing Z-DEVD-R110 substrate was added to the cell lysate, with a subsequent incubation at room temperature for 45 minutes. The rhodamine 110-derived substrate (Z-DEVD-R110) used in this assay is a non-fluorescent bisamide compound that, upon enzymatic cleavage via active caspase 3 and maybe caspase 7 in the cell lysates, is converted in a two-step process to the fluorescent monoamide and then to the even more fluorescent R110 product. Both of these products were then measured using a Biotek synergy 2 fluorescent plate reader at corresponding wavelengths (excitation 496 nm/emission 520 nm). For our experiments, the fluorescent reading was normalized to the amount of protein in the cell lysate as determined via standard BCA assay (Pierce Thermo Fisher Scientific). Relative fluorescence was calculated after setting the no-treatment control to 1.

### Western blot analysis

Around 10 million cells were spun down at 200 rcf for 5 minutes and rinsed once with PBS before lysing in 50 μl of Millipore Milliplex MAP lysis buffer supplemented with protease and phosphatase inhibitors (Roche). Protein denatured in 4X sample buffer supplemented with β-mercaptoethanol (Bio-Rad) was loaded per well. Bio-Rad stain-free Criterion 4-20% precast gels were used. After running the gel at 140 volts for 90 minutes, Bio-Rad gel imager was used to activate the stain-free technology to visualize the total protein levels loaded in the gel. A nitrocellulose turbo-transfer pack and system (Bio-Rad) was then used to transfer the proteins to the membrane, and 5% w/v dry milk in Tris-buffered saline-0.1% Tween 20 (TBS-T) was used to block the membranes for 1 hour. Membranes were then incubated with the primary antibody diluted in 5% BSA in TBS-T overnight. Membranes were then rinsed in TBS-T 3 times (one 15 minute wash and two 5 minutes washes) and then incubated with the corresponding secondary antibody-conjugated with HRP for 1 hour and rinsed with TBS-T as before. After the final wash, membranes were developed using a Pierce SuperSignal West Pico chemiluminescence kit and visualized using the Bio-Rad imaging system. Antibodies used included: Rabbit anti-GSK-3β (Cell Signaling, Cat. No: 12456), Rabbit anti-phospho-GSK-3β (Y216) (Abcam, Cat. No: ab75745), Rabbit anti-c-MYC (Cell Signaling, Cat. No: 5605), Rabbit anti-phospho-c-MYC (Ser 62) (Cell Signaling, Cat. No: 13748), Rabbit anti-phospho-c-MYC (Thr58) (Abcam, Cat. No: ab185655), and Mouse anti-β-Actin (Sigma, Cat. No: A5441) at a 1:1000 dilution. Anti-Rabbit HRP and Anti-Mouse HRP secondary antibodies were purchased from Cell Signaling and used at a 1:5000 dilution. When necessary, membranes were stripped using RESTORE PLUS Western blot stripping buffer (Pierce) for 10 mins and washed with TBS-T several times and re-blocked and re-probed as before. Quantification of the band intensities were performed using Image J software (NIH).

### Luminex analysis

Signaling changes in NF-κB [MILLIPLEXMAP NF-κB Signaling Magnetic Bead Kit 6-plex Kit, EMD Millipore, analytes: c-MYC, FADD (Ser194), IκBα (Ser32), IKKα/β (Ser177/Ser181), NF-κB (Ser536), TNFR1], DNA damage [MILLIPLEX MAP DNA Damage/Genotoxicity Magnetic Bead Panel, EMD Millipore, analytes: ATR (total), Chk1 (Ser345), Chk2 (Thr68), H2A.X (Ser139), MDM2 (total), p21 (Total), p53 (Ser15)], and apoptotic pathways [Bio-plex pro RBM apoptosis panel 2 and 3, Bio-Rad, analytes: Bad, Bax/Bcl-2 dimer, Bcl-xL, Bim, Mcl-1, active caspase 3, Bcl-xL/Bak dimer, Mcl-1/Bak dimer, survivin] associated with 1 μM 9-ING-41 treatment for 48 hours as compared to no-treatment controls were determined using Luminex multiplex technology with a FLEXMAP 3D instrument, as per manufacturer instructions. Cells were lysed in MILLIPLEX MAP Lysis buffer supplemented with protease inhibitor cocktail (Sigma) and phosphatase inhibitor cocktails 2 and 3 (Sigma) and, after BCA protein determination, 15 μg of protein was added to each well. All samples were run in duplicate and changes in MFI or absolute quantity between 9-ING-41-treated cells and non-treated control were analyzed, and an unpaired t-test was performed to determine statistical significance.

### Dose response series for combinatorial strategies

Daudi, SUDHL-4, KPUM-UH1, Karpas 422, or TMD8 cells were treated simultaneously with a series of concentrations of 9-ING-41 (0 μM, 0.05 μM, 0.5 μM) and either Venetoclax (0 nM, 0.5 nM, 5 nM, 50 nM, 500 nM, 5000 nM), BAY-1143572 (0 μM, 0.005 μM, 0.05 μM, 0.5 μM, 5 μM, 50 μM), or Idelalisib (0 μM, 0.005 μM, 0.05 μM, 0.5 μM, 5 μM, 50 μM). Viability at day 3 using an MTS assay was determined as above. The background absorbance was subtracted from the A490 of the samples and the A490 of the vehicle/no-treatment control was set to 1, and the relative A490s of the rest of the samples were calculated. The IC50 was calculated as the concentration of the drug at which A490 reached 0.5. Additive effects were calculated as a fold change in the IC50 of a novel agent when combined with 0.5 μM of 9-ING-41. Synergistic effects were explored using the Chou-Talay method with Compusyn software but could not be reliably interpreted due to 9-ING-41 not reaching IC50 for some cell lines (peak inhibition of 40%) and resultant use of predicted values for synergy calculations.

## SUPPLEMENTARY MATERIALS FIGURES



## Abbreviations

GSK: Glycogen synthase kinase
DLBCL: diffuse large B-cell lymphoma
CDK: cyclin-dependent kinase
DHL: double-hit lymphoma
ALL: acute lymphoblastic leukemia
