# Supplementary material for: GSK-3β inhibitor, 9-ING-41, reduces cell viability and halts proliferation of B-cell lymphoma cell lines as a single agent and in combination with novel agents
Source: Oncotarget. 2017 Nov 11;8(70):114924–34. doi: 10.18632/oncotarget.22414 (PMC5777742; doi:10.18632/oncotarget.22414)
Supplement: Supplementary file 1 [file oncotarget-08-114924-s001.pdf]

GSK-3β inhibitor, 9-ING-41, reduces cell viability and halts proliferation of B-cell lymphoma cell lines as a single agent and in combination with novel agents

SUPPLEMENTARY MATERIALS

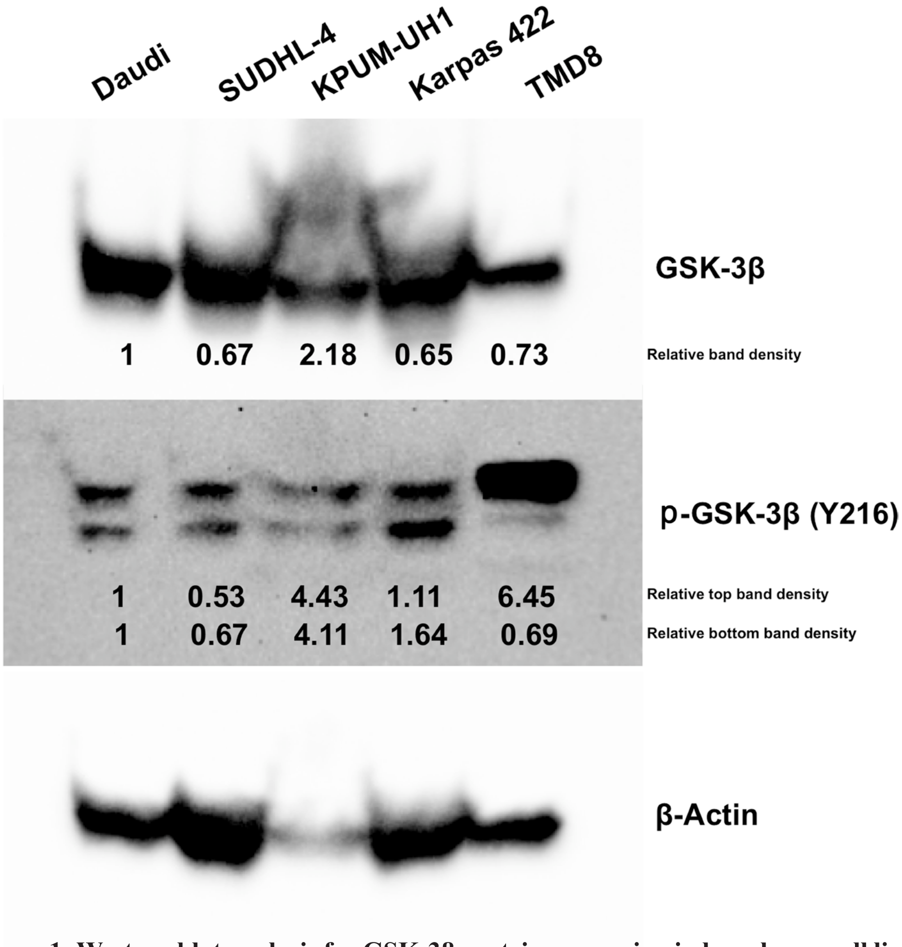

Supplementary Figure 1: Western blot analysis for GSK-3β protein expression in lymphoma cell lines. Daudi, SUDHL-4, KPUM-UH1, Karpas 422 and TMD8 cells were lysed and analyzed for total GSK-3β, p-GSK-3β (Y216) and stripped and re-probed for β-Actin (loading control) as described in materials and methods. The band intensities were calculated using Image J (NIH) software and normalized to β-actin control, and the relative values for all cell lines compared to Daudi is shown. Note that p-GSK-3β (Y216) has 2 bands, and the quantification is shown for both. However, the top band may represent the α-isoform due to the possibility of antibody cross-reactivity and has not been verified.

A.

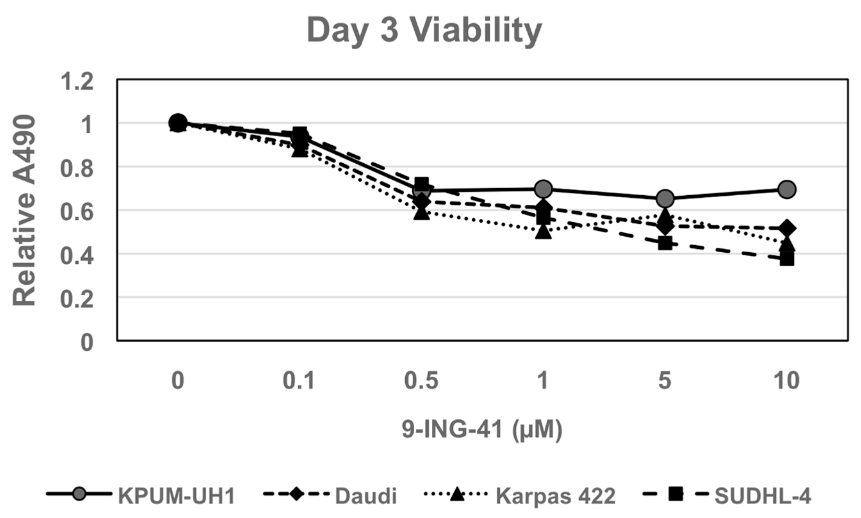

B.

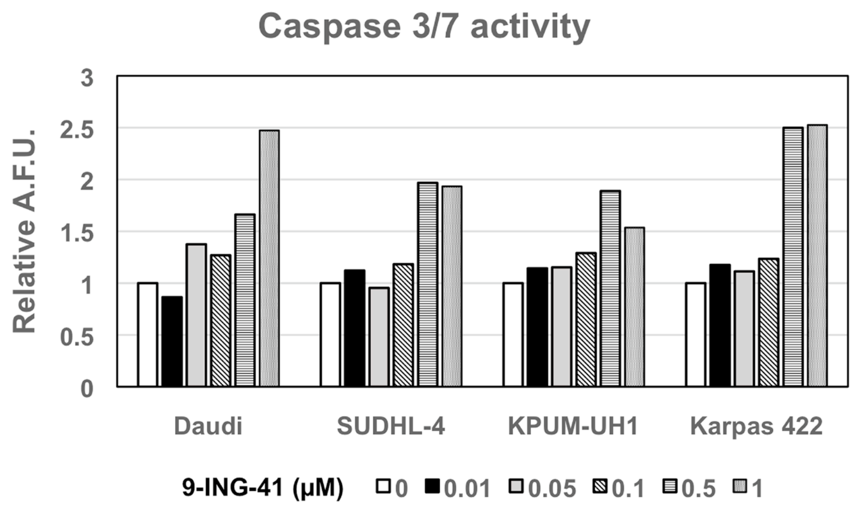

Supplementary Figure 2: Viability and caspase activity in cells treated with varying concentrations of 9-ING-41. (A) 50,000 cells were plated in 96-well plate wells and treated with varying concentrations of 9-ING-41. Seventy two hours later, cell viability was determined using, MTS assay. Briefly, 20µl of MTS reagent was added to cells and incubated for 2 hours and, absorbance at 490 nm was read using a Biotek plate reader. Relative absorbance is calculated after setting the average absorbance of the no-treatment control as 1. **(B)** 100,000 cells were plated in 12-well plate wells in duplicate and treated with varying concentrations of 9-ING-41. Twenty four hours later, cells were lysed and analyzed for caspase 3/7 activity as described in materials and methods. The average of the duplicate is shown in the graph.

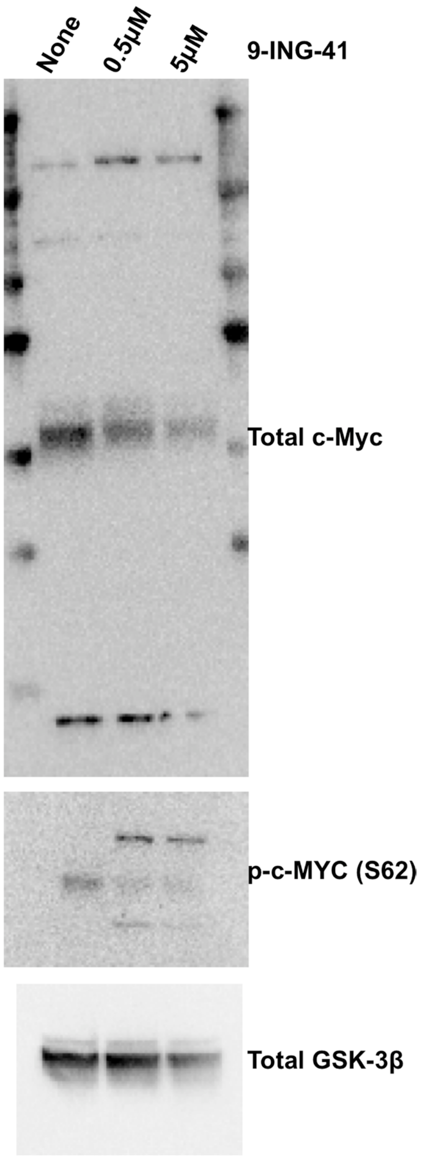

Supplementary Figure 3: Western blot analysis for c-MYC protein expression in the KPUM-UH1 cell line treated with 9-ING-41. KPUM-UH1 cells were left untreated or treated with 0.5 or 5 μM 9-ING-41 for 24 hours and lysed and analyzed for total c-MYC, p-c-MYC (Ser62) and total GSK-3β as before. Note the additional bands seen in 9-ING-41-treated conditions for p-c-MYC (Ser 62). The extreme lanes on the right and left are loaded with protein ladder and have cross-reactivity to the c-MYC antibody.

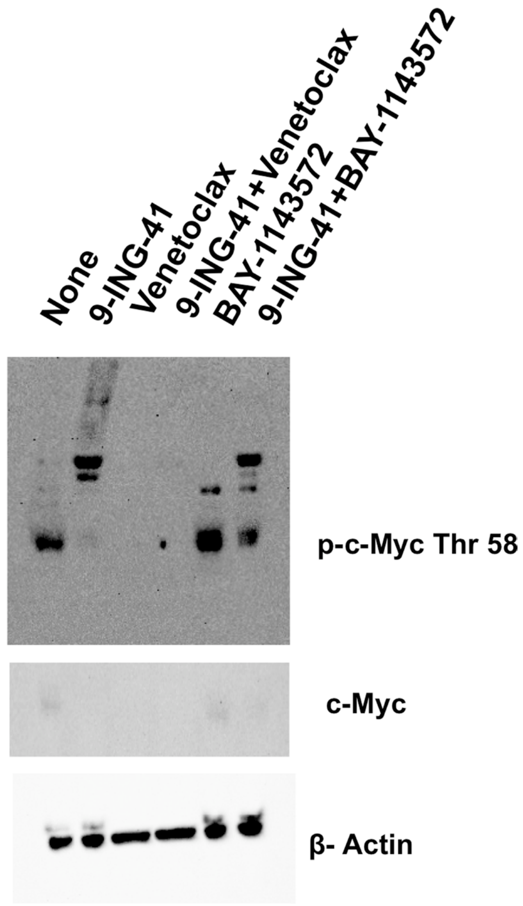

Supplementary Figure 4: Western blot analysis for c-MYC protein expression in the KPUM-UH1 cell line treated with 9-ING-41 in combination with Venetoclax or BAY-1143572. KPUM-UH1 cells were left untreated or treated with the following: 0.5uM 9-ING-41; 10nM Venetoclax; the combination of 9-ING-41 and Venetoclax; 1uM BAY-1143572; or the combination of 9-ING-41 and BAY-1143572 for 48 hours and lysed and analyzed for total c-MYC, p-c-MYC (Thr58) and β-Actin as before. Note the additional bands seen in 9-ING-41-treated conditions for p-c-MYC (Thr58).
